# SMS Text Messages for Parents for the Prevention of Child Drowning in Bangladesh: Acceptability Study

**DOI:** 10.2196/16958

**Published:** 2020-09-23

**Authors:** Md Mosharaf Hossain, Kulanthayan Mani, Ruhani Mat Min

**Affiliations:** 1 Faculty of Business, Economics and Social Development Universiti Malaysia Terengganu Kuala Nerus, Terengganu Malaysia; 2 Safe Kids Malaysia and Department of Community Health Faculty of Medicine and Health Sciences Universiti Putra Malaysia Selangor Malaysia

**Keywords:** acceptability, SMS, drowning, parents

## Abstract

**Background:**

In many cases, greater use is being made of mobile phone text messages as a means of communication between patients and health care providers in countries around the world.

**Objective:**

We studied the use of mobile phones and the factors related to the acceptability of text messages for parents for the prevention of child drowning in Bangladesh.

**Methods:**

From a randomized controlled trial involving 800 parents, 10% (80/800) were selected, and socioeconomic status, mobile phone use, and acceptability of SMS text messages for drowning prevention were measured. Participants with at least one child under 5 years of age were selected from rural areas in Rajshahi District in Bangladesh. Mobile phone–based SMS text messages were sent to the participants. Multivariate regression was used to determine the factors related to the acceptability of text messages for the prevention of child drowning in Bangladesh.

**Results:**

The acceptability of SMS text messages for the prevention of child drowning in Bangladesh was significantly lower among women (odds ratio [OR] 0.50, 95% CI 0.12-1.96, *P*=.02) than among men, lower for parents older than 30 years (OR 0.17, 95% CI 0.14-1.70, *P*=.01) compared to parents younger than 30 years, higher among parents who had an education (OR 1.63, 95% CI 1.11-5.80, *P*=.04) than among illiterate parents, and higher among parents with a monthly household income over 7000 Bangladeshi Taka (approximately US $82.54; OR 1.27, 95% CI 1.06-1.96, *P*=.05) than among parents whose monthly income was less than 7000 Bangladeshi Taka.

**Conclusions:**

The high percentage of mobile phone use and the acceptability of SMS text messages for parents for the prevention of child drowning are encouraging, in terms of identifying the best strategy for using such technologies, and deserve further evaluation.

## Introduction

Water plays an important role in children’s daily life. They like to play with water, have fun, and sometimes are more adventurous. Children are always excited around water, no matter whether it is a pond, pool, lake, in an open field, or simply on or beside the road after rain falls. It is impossible for children to grow up without water, as besides playing, they need it to clean themselves, and they gain comfort and are cooled by it. Although water is considered to be an important element for children to survive, it is also a hazard if there is a lack of awareness concerning the dangers it presents [1].

A small child can drown in only a few inches of water—under a bucket, in a field, or in the bathtub. Drowning injuries have an epidemiological pattern. However, the pattern changes depending on the type of water body, age group, and activity. Drowning ranks among the top 3 causes of accidental deaths in most countries around the world, and the mortality rates are highest in children under 5 years of age [1].

Drowning is the leading cause of death for children aged 1 year and over in Bangladesh. According to the rate reported in the Bangladesh Health and Injury Survey [2], nearly 17,000 children drowned in 2004, approximately 46 per day, and close to 4 times more than the rate in other low-income countries [2]. This equates to approximately 188 children drowning a day. In Bangladesh, drowning rates are 10 to 20 times higher than those in high-income countries [2]. There is a question concerning whether drowning is always fatal or whether it is possible to survive. While many people think that drowning is always a fatal event, others think that near drowning is a result that is never lethal [2].

As a result, drowning injuries are now the leading cause of death, disability, and severe morbidity among children in low- and middle-income countries in Asia, such as Bangladesh. This compromises the gains that were previously achieved, at high cost, to prevent other causes of illness and injury among children, and it jeopardizes the continued progress in survival and security. One conclusion is that drowning injuries are so important that they must be treated first [3].

Drowning reduces the impact of other interventions for children, as children who died from drowning often received conventional vaccines, vitamin A supplementation, and other food aid. They often used early development and education programs, and such investments are lost when a child drowns. Effective drowning prevention interventions are available at all stages of childhood and are cost-effective alongside traditional interventions for children [2].

Mobile phones and SMS text messages are gradually being used between patients and their health care providers in many countries around the world because of their communication potential [4]. Several studies [5-7] have shown that SMS text message–based counseling and observation can restore patient behavior and health outcomes. However, the potential use of SMS text messages in clinical settings in low-income countries has not been well established. The feasibility of implementing such technologies is not clearly demonstrated, nor is there a cost-effective and sustainable payment mechanism or commercial model that can be improved [8-12]. Information concerning how patients appreciate or perceive the use of SMS text messages from a mobile phone to improve disease management can be valuable in implementing such methods in primary care. Therefore, this study aimed to determine the access to mobile phones and the willingness to receive SMS text messages on a mobile phone; and determine the factors related to the willingness to receive mobile phone–based SMS text messages concerning child drowning prevention.

## Methods

### Overview

The researchers focused on the acceptability of text messages for child drowning prevention as part of a randomized controlled trial (International Standard Randomized Controlled Trial Number; ISRCTN13774693) of a mobile phone–based SMS text messages intervention aimed at improving the knowledge, attitude, and practices of parents about child drowning in Bangladesh. As part of a larger study, the design of the study and the data collection procedure followed the protocol of a previously published randomized controlled trial [13]. The sample size was 80 parents from 2 villages (10% of the randomized controlled trial's sample size). The parents received an SMS text message before the interview.

The mobile phone–based SMS text message intervention aimed to improve parents' knowledge, attitudes, and practices in relation to the prevention of child drowning in Bangladesh. The researcher developed the SMS text messages based on focus group discussions and literature reviews. The messages used informal language and were sent every Friday. On Friday morning, each week, the research team sent out SMS text messages to parents in the intervention group. The message was typically 150-200 characters long and in the Bangla language.

Data were collected through face-to-face interviews in rural areas using a structured questionnaire, which was conducted by a team of 2 qualified research assistants. The inclusion criteria were the same as those for the published study protocol [13]. Parents meeting the selection criteria were included in the study. The questionnaires included questions on socioeconomic characteristics (age, sex, marital status, education, and income), mobile phone use, SMS text message use, and drowning knowledge.

### Data Analysis

The researchers performed chi-square tests to assess the importance of the responses and associated factors regarding SMS text messaging concerning drowning in childhood. A univariate logistic regression analysis was used to evaluate the relationship between SMS text message reading and the individual variables. Factors having a statistically significant association with the reading of text messages on child drowning in univariate analysis (*P* value<.25) were included in the final logistic model (*P* value<.05) taking into account other variables. SPSS statistical software (version 22; IBM Corp) was used for data analysis. A value of *P*<.05 was considered statistically significant.

### Ethical Statement

The ethical approval for this study was obtained from University Putra Malaysia (UPM/TNCPI/RMC/1.4.18.1 (JKEUPM)/F2) and the Centre for Injury Prevention and Research, Bangladesh. Informed written agreements were obtained from all the respondents before the data were collected. All the respondents were assured that the data would only be used for research purposes and that all the answers would be kept confidential.

## Results

The association between the acceptability of text messages about childhood drowning and the selected sociodemographic characteristics of parents are presented in Table 1. Table 1 reveals the associations between parent age, gender, educational status, occupation, monthly income, the ability to read SMS text messages, liking SMS text messages, and having Bangla language on their phone with the acceptability of text messages on childhood drowning. The acceptability of text messages concerning childhood drowning prevention was significantly higher among males (76%, χ^2^=4.39, *P*<.001), parents aged less than 30 years (82%, χ^2^=6.54, *P*<.001), literate parents (76%, χ^2^=4.98, *P*<.001), parents with a monthly income of more than 7000 BDT (80%, χ^2^=5.69, *P*<.001), parents who had the ability to read an SMS text messages (71%, χ^2^=3.94, *P*<.001), parents who liked mobile phone–based SMS text messages (74%, χ^2^=7.51, *P*<.001), and parents who had Bangla on the mobile phone (73%, χ^2^=5.63, *P*<.001) (Table 1). There were no significant associations between parents’ occupation, type of mobile phone, use of mobile phone, knowledge about drowning with the acceptability of text messages on childhood drowning.

**Table 1:** The results of chi-square tests concerning the acceptability of text messages about childhood drowning among various sociodemographic variables in Bangladesh.

Variables		All (N=80), n (%)	Acceptability		Comparison	
			No (n=26), n (%)	Yes (n=54), n (%)	Chi-square (*df*)	*P* value
**Age (years)**					6.54 (1,80)	<.001
	Below 30	38 (47)	7 (18)	31 (82)		
	More than 30	42 (53)	19 (45)	23 (55)		
**Gender**					4.39 (1,80)	<.001
	Male	50 (63)	12 (24)	38 (76)		
	Female	30 (37)	14 (47)	16 (53)		
**Educational status**					4.98 (1,80)	<.001
	Illiterate	30 (37)	14 (47)	16 (53)		
	Literate	50 (63)	12 (24)	38 (76)		
**Occupation**					0.97 (1,80)	.563
	Housewife/Farmer	66 (83)	21 (38)	38 (62)		
	Other	14 (17)	5 (17)	16 (83)		
**Monthly income (BDT^a^)**					5.69 (1,80)	.001
	Less than 7000	40 (50)	8 (20)	22 (55)		
	More than 7000	40 (50)	18 (45)	32 (80)		
**Phone type**					0.38 (1,80)	.781
	Normal	68 (85)	11 (37)	19 (63)		
	Smartphone	12 (15)	15 (30)	35 (70)		
**Phone use**					0.36 (1,80)	.144
	Father	30 (37)	21 (38)	45 (66)		
	Mother	50 (63)	5 (29)	9 (71)		
**Ability to read SMS text messages**					3.94 (1,80)	<.001
	No	10 (12)	6 (50)	4 (50)		
	Yes	70 (88)	20 (29)	50 (71)		
**Liked SMS text messages**					7.51 (1,80)	<.001
	No	12 (15)	8 (66)	4 (34)		
	Yes	68 (85)	18 (26)	50 (74)		
**Have Bangla language on phone**					5.63 (1,80)	<.001
	No	11 (14)	7 (63)	4 (37)		
	Yes	69 (86)	19 (27)	50 (73)		
**Knowledge about drowning**					3.94 (1,80)	.346
	No	10 (12)	6 (50)	4 (50)		
	Yes	70 (88)	20 (29)	50 (71)		

^a^BDT: Bangladeshi Taka; an exchange rate of US $1 to 0.012 BDT is applicable.

**Figure 1:**
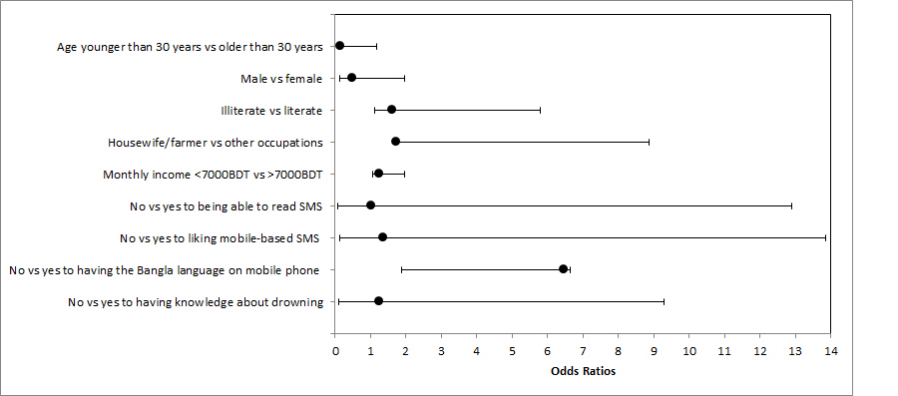
Forest plot of acceptability of text messages for parents on childhood drowning prevention odds ratios.

In the logistic analysis (Table 2), the acceptability of text messages on childhood drowning was significantly higher among males. The odds ratio (OR) of 0.50 for females was less than 1, indicating that female respondents considered text messages on childhood drowning to be 0.50 (adjusted OR 0.50, *P*=.02, 95% CI 0.12-1.96) times less acceptable than their male counterparts.Parents aged more than 30 years considered text messages on childhood drowning to be 0.17 times less acceptable than parents aged less than 30 years (OR 0.17, *P*=.01, 95% CI 0.14-1.70). Parents who had an education considered text messages on childhood drowning to be 1.63 times (OR 1.63, *P*=.04, 95% CI 1.11-5.80) less acceptable than parents who had no education, those with a household income of more than 7000 BDT considered text messages on childhood drowning to be 1.27 times more acceptable than those with a household income of less than 7000 BDT (OR 1.27, *P*=.05, 95% CI 1.06-1.96), and parents who had Bangla on their mobile phone considered text messages on childhood drowning to be 6.46 times more acceptable than parents who did not have the Bangla language on their mobile phone (OR 6.46, *P*=.05, 95% CI 1.89-6.65) (Figure 1). There were no significant associations among parents’ occupation (OR 7.92, *P*=.052, 95% CI 0.97-64.33) or knowledge about drowning (OR 3.73, *P*=.05, 95% CI 0.95-14.71) with the acceptability of text messages on childhood drowning.

**Table 2:** Results of the logistic analysis of the acceptability of text messages on childhood drowning among various sociodemographic variables.

Variable^a^		OR^b^	*P* value	95% CI	Adjusted OR	*P* value	95% CI
**Age (years)**							
	Below 30^c^	1	—	—	1	—	—
	More than 30	0.27	<.001	0.09-0.75	0.17	<.001	0.14-1.70
**Gender**							
	Male^c^	1	—	—	1	—	—
	Female	0.36	.06	0.13-0.94	0.5	.02	0.12-1.96
**Educational status**							
	Illiterate^c^	1	—	—	1	—	—
	Literate	2.77	.03	1.05-7.29	1.63	.04	1.11-5.80
**Occupation**							
	Housewife/Farmer^c^	1	—	—	1	—	—
	Other	7.92	0.052	0.97-64.33	1.73	.16	1.71-8.87
**Monthly income (BDT^d^)**							
	Less than 7000^c^	1	—	—	1	—	—
	More than 7000	1.30	<.001	0.98-1.82	1.27	.06	1.06-1.96
**Ability to read SMS text messages**							
	No^c^	1	—	—	1	—	—
	Yes	3.75	.05	0.95-14.71	1.04	.98	0.08-12.91
**Liked SMS text messages**							
	No^c^	1	—	—	1	—	—
	Yes	5.55	<.001	1.49-20.70	1.38	.78	0.13-13.87
**Have Bangla language on phone**							
	No^c^	1	—	—	1	—	—
	Yes	4.6	.02	1.20-17.53	6.46	.053	1.89-6.65
**Knowledge about drowning**							
	No^c^	1	—	—	1	—	—
	Yes	3.73	.05	0.95-14.71	1.26	.99	0.10-9.30

^a^ Model Summary—2-loglikelihood: 71.12; Cox & Snell R Square: 0.31; Negelikereke R: 0.43; model chi-square: 29.77.

^b^OR: odds ratio.

^c^Reference.

^d^BDT: Bangladeshi Taka; an exchange rate of US $1 to 0.012 BDT is applicable.

## Discussion

### Principal Findings

The aim of this research was to identify access to mobile phones, the willingness to receive mobile phone–based SMS text messages in relation to the prevention of childhood drowning, and associated factors among parents of children under 5 in the rural areas of Bangladesh. The results of this study show that there is high (54/80, 68%) mobile phone access among parents with children less than 5 years of age. Of the respondents who had access to mobile phones, the majority (59/80, 74%) would like to receive phone-based SMS text messages. This is similar to the findings of a study conducted in northwest Ethiopia concerning mobile health services among patients with diabetes by Jemere et al [14] in which 71% were willing to receive mobile phone–based health services.

Age, gender, educational status, monthly income, ability to read SMS text messages, liking mobile phone–based SMS text messages, and having Bangla language software on their mobile phone were associated with the acceptability of text messages on childhood drowning prevention in Bangladesh. The majority (58/80, 72%) were able to read SMS text messages. In the study, those in the age group of less than 30 years showed a higher acceptability of receiving text messages on childhood drowning than those in the other age group (≥30 years); the acceptability of text messages on childhood drowning decreased slightly with age. Older respondents were less likely to possess the technology and knowledge compared to their younger counterparts, which could be the reason for the lower acceptability of text messages concerning childhood drowning. This is similar to the findings of a study in Bangladesh conducted by Islam et al [15] in which all the participants owned a mobile phone and about half of the participants reported being able to retrieve and read SMS text messages.

Among males, only those with a household income above 7000 BDT, and parents who had the Bangla language on their mobile phone were associated with the acceptability of text messages on childhood drowning prevention. An increase in education is associated with an increase in the proportion of income earners per household and had an impact on the acceptability of text messages on childhood drowning prevention, which is contrary to the assertions in the literature in this regard [15]. Those with a relatively higher income had greater willingness to read text messages on childhood drowning prevention, which is aligned with the expectations [15]. The association is likely to be due to the fact that a higher educational status is likely to lead to improved knowledge and awareness of drowning, and better access to mobile phone and mobile phone networks. Awareness concerning how to apply these new technologies to consumer health and enable patients to take control and play an active role in managing their health has increased. Consumer health interventions have been used, for example, to help people monitor their own health [16], provide social information and support, and remotely monitor their home [17]. Many studies [18-20] have used mobile phone technology for public health issues and have commented on other considerations for older people in terms of design, usability and functionality. Studies [18] have shown that interventions need to take into account psychological assumptions or barriers for use among older people.

Kotani et al [19] noted the success of using mobile phones to take pictures, even when older people refused to take pictures with traditional cameras. Mobile phones and telephones were perceived as informal and ubiquitous and were therefore considered acceptable while traditional cameras were not [20].

Mobile phone interventions for the health of the elderly are in their infancy and are just beginning to develop. The rapid development of mobile phones, coupled with the rapid aging of the population, provides an excellent opportunity to use mobile phone technology to better manage the health of seniors and positively impact their quality of life and well-being. Early childhood research in the field allow interested researchers to conduct research in all directions while pursuing the same goal of improving the lives of older people [21,22]. The prevalence of mobile phones, text messages, and in particular, the growing use of instant messengers and social networking apps in health care highlight ways to significantly integrate health care into users' daily activities instead of artificially complementing health care processes [23].

There are clear benefits from these interventions, such as further educating parents about their treatment, providing more opportunities for health care professionals and parents to interact efficiently and effectively, and improving health literacy in general. Directed and monitored interventions also provide end users with reliable information in a condensed and easily accessible form. More research is required to optimize the use of these interventions, with attention particularly focused on sensitive areas, such as cost and time burden for the providers and consumers of mobile phone health care services. The study participants in this trial mostly had (more than 87%) access to a mobile phone, which might be different from other populations in Bangladesh [24-27].

### Strengths and Limitations

This study provides quality evidence and establishes an association between the intervention and outcomes of the study in Bangladesh among parents of children aged under 5 years old.

These findings are consistent with the results reported by several researchers in similar studies [13-15] around the world concerning different injuries and provide information that is critical for controlling the drowning epidemic, especially in low- and middle-income countries that are highly burdened by such events.

The mobile phone intervention used in this study appears to be a relatively cheap and acceptable strategy for improving the drowning prevention knowledge, attitude, and practices of parents of children under 5 years old. The data concerning the cost-effectiveness of this strategy in comparison to those of other interventions were not reported because it was beyond the scope of this study.

### Conclusion

The results of this study show that the vast majority of parents in rural areas in Bangladesh find SMS text messages to prevent drowning acceptable. Given that the number of drowning deaths is still high and the low cost of mobile phones in Bangladesh, a self-sustaining business model in low-income countries and other middle-income countries is possible. The age of the respondents, gender, educational status, monthly income, ability to read SMS text messages, liking mobile phone–based SMS text messages, and Bangla language software on the mobile phone were associated with the acceptance of text messages for preventing drowning. Based on this outcome, mobile phone SMS text messages implementation, such as self-monitoring, behavioral counseling, and interventions can be used to improve knowledge, attitudes, and practices.

## Abbreviations

OR: odds ratio
